# The Erythropoetin *rs1617640* Gene Polymorphism Associates with Hemoglobin Levels, Hematocrit and Red Blood Cell Count in Patients with Peripheral Arterial Disease

**DOI:** 10.3390/genes11111305

**Published:** 2020-11-04

**Authors:** Wilfried Renner, Melanie Kaiser, Sebastian Khuen, Olivia Trummer, Harald Mangge, Tanja Langsenlehner

**Affiliations:** 1 Clinical Institute of Medical and Chemical Laboratory Diagnostics, Medical University of Graz, Auenbruggerplatz 15, 8036 Graz, Austria; wilfried.renner@medunigraz.at (W.R.); melanie.kaiser@medunigraz.at (M.K.); sebastian.khuen@medunigraz.at (S.K.); 2 Division of Endocrinology, Department of Internal Medicine, Medical University of Graz, 8036 Graz, Austria; olivia.trummer@medunigraz.at; 3 Department of Therapeutic Radiology and Oncology, Medical University of Graz, 8036 Graz, Austria; tanja.langsenlehner@medunigraz.at

**Keywords:** peripheral arterial disease, genetics, epidemiology, erythropoietin

## Abstract

Background: Erythropoietin has a pivotal role in erythropoiesis and angiogenesis. A common polymorphism (rs1617640, A > C) in the promoter of the erythropoietin gene (EPO) has been associated with erythropoietin expression and microvascular complications of diabetes. We aimed to analyze the potential role of this polymorphism in the pathogenesis of peripheral arterial disease (PAD). Methods: EPO genotypes and laboratory markers for erythropoiesis were determined in 945 patients with PAD. Results: The minor EPO *rs1617640* C-allele was associated in an allele-dose-dependent manner with hemoglobin levels (*p* = 0.006), hematocrit (*p* = 0.029), and red blood cell count (*p* = 0.003). In a multivariate linear regression analysis including conventional risk factors diabetes, sex, and smoking, EPO genotypes were furthermore associated with age at onset of PAD symptoms (*p* = 0.009). Conclusions: The EPO *rs1617640* gene polymorphism affects erythropoiesis, leads to an earlier onset of PAD, and is a potential biomarker for the pathogenesis of this disease.

## 1. Introduction

Peripheral arterial disease (PAD) is a chronic progressive disease resulting from reduced blood flow to tissues of the legs, usually caused by atherosclerosis of leg arteries [1,2]. Main risk factors for PAD are smoking, diabetes, hypertension, and hyperlipidemia, but additional independent genetic risk factors may contribute to the development of PAD. After adjustment for classical risk factors, the heritability of PAD is estimated to be around 20% [3,4,5].

Erythropoietin is a glycoprotein hormone with the main function of stimulating erythropoiesis [6]. The gene for erythropoietin, EPO, is activated under hypoxic conditions by binding of hypoxia inducible factors 1α and 1β to the EPO promoter [7]. Beyond its pivotal role in erythropoiesis, erythropoietin has been shown to be a potent angiogenic factor [8,9]. Angiogenesis, the growth of new capillaries from the existing vascular structures, is triggered by endothelial cell proliferation and migration. This process may lead to the formation of a collateral circulation functioning as “endogenous bypass vessels”. In early stages of PAD, the collateral circulation system is beneficial on the symptoms and outcomes of PAD and may at least delay the onset of clinical symptoms of the disease [10,11,12]. In patients with chronic total occlusion of coronary vessels, increased serum erythropoietin levels were a positive predictor of good collateral development [13].

In 2008, Tong and coworkers reported that a functional polymorphism in the promoter of the EPO gene (*rs1617640*, c.-1306A>C) is associated with erythropoietin expression and erythropoietin levels [14]. In vitro luciferase reporter expression of the minor *rs1617640* C-allele was 25-fold decreased compared to the common A allele, and erythropoietin concentration in vitreous samples of subjects with the CC genotype were 7.5-fold lower compared to those of subjects with the AA genotype [14]. In patients with chronic hepatitis C receiving antiviral treatment, homozygosity for the EPO *rs1617640* CC genotype was associated with a weaker rise in erythropoietin levels and a more frequent need for blood transfusion [15]. In a recent large trans-ethnic genome-wide association study, the EPO rs1617640 polymorphism was significantly (*p* < 5 × 10^−9^) associated with red blood cell count [16].

In contrast to these findings, Fan et al. reported that the C-allele was associated with higher erythropoietin concentrations in an allele-dose-dependent manner [17]. Similarly, Khabour et al. found a higher frequency of the C-allele in blood donors with upper limit hematocrit levels [18] and Kästner et al. showed the C-allele to be associated with higher EPO promoter activity [19]. These different findings indicate that the effects of the EPO *rs1617640* polymorphism on erythropoietin production may depend on physiological and pathological conditions.

The primary aim of the current study was to elucidate the potential association of EPO *rs1617640* genotypes with markers for erythropoiesis in patients with PAD.

## 2. Materials and Methods

### 2.1. Human Subjects

Patients of the current study have been described previously [20,21]. Briefly, we recruited 951 patients with PAD between 1997 and 2000 at the Division of Angiology at Department of Internal Medicine, Medical University of Graz, Austria. All patients underwent physical examination, a detailed clinical interview, ankle and brachial systolic pressure measurement with a Doppler ultrasound device, and vascular assessment of the leg arteries by duplex scanning. Definition of PAD was the presence of >50% stenosis of the lower limb artery or an ankle–brachial index (ABI) < 0.9. Disease stage was determined according to Fontaine classification (II: intermittent claudication; III: rest pain; IV: gangrene). From six PAD patients, no samples for genotyping were available and these patients were excluded from the present study. The remaining 945 patients formed the study population.

The study was approved by the Ethical Committee of the Medical University of Graz (ethical vote 09-124 ex 98/99) and written informed consent was obtained from all participating subjects. The study followed the rules of the Declaration of Helsinki. All subjects were of Caucasian ethnicity.

### 2.2. Clinical Examination and Laboratory Methods

Cardiovascular risk factors and previous or current cardiovascular disease were identified using self-reported medical and medication history, medical records from the Medical University Graz, and medical records provided by general practitioners. Ankle pressure was measured at the posterior tibial artery and calculation of ABI was performed according to Sanchez and Veith [22]. Smoking habit as well as age at first onset of PAD were asked in a face-to-face interview. Diabetes was diagnosed using the World Health Organization criteria [23]. Blood samples for laboratory analyses were drawn in the morning after an overnight fast. Hemoglobin levels, hematocrit, and red blood cell count values were available for 887 (93.9%) patients.

Genomic DNA was isolated from anticoagulated whole blood on a MagNA Pure instrument (Roche, Vienna, Austria) and genotyping was performed using the 5′-exonuclease (TaqMan) technology as described previously [24]. To verify the accuracy of genotyping, analysis of 96 samples was repeated and no discrepancies were found.

### 2.3. Statistics

Genotype Hardy–Weinberg equilibrium was analyzed using the HW Diagnostics software (Fox Chase Cancer Center, Philadelphia, PA, USA, 1999). IBM SPSS Statistics release 23 was used for statistical analyses. Continuous variables were analyzed by ANOVA and presented as means ± standard deviation (SD). Categorical variables are presented as percentages and were compared by chi-square test or Kruskal–Wallis test. For multivariate regression analyses, EPO genotypes were coded assuming an allele dose-effect (wild-type genotype = 0, heterozygous carrier of the minor allele = 1, homozygous carrier of the minor allele = 2). The criterion for statistical significance was *p* < 0.05.

## 3. Results

Demographic data and EPO genotypes of study subjects are summarized in Table 1. Genotypes were determined successfully in all PAD patients and did not deviate from the Hardy–Weinberg equilibrium. EPO genotypes were not associated with classical PAD risk factors diabetes, hypertension, hypercholesterolemia, smoking history, or male sex. The minor EPO *rs1617640* C-allele was associated in an allele-dose-dependent manner with higher hemoglobin levels, hematocrit, and red blood cell count (Table 2).

Thirty-five patients (3.7%) were unable to specify their age at onset of PAD. In the remaining 910 patients, mean age at onset of PAD was 64.8 ± 11.1 years. In a crude univariate regression analysis, EPO genotypes were associated with age at onset of PAD (regression coefficient −1.17; *p* = 0.025). In a multivariate linear regression analysis including classical PAD risk factors, EPO genotype remained a significant predictor of age at onset of PAD (Table 3). Each additional minor EPO *rs1617640* C-allele accounted for a 1.3 year earlier onset of PAD.

## 4. Discussion

Herein, we report that a functional genetic variation in the EPO promoter is associated with higher hemoglobin levels, hematocrit, and red blood cell count. These three common laboratory markers are well-established indicators of erythropoiesis. Although we did not evaluate the biological mechanism for the observed association in this study, it is likely that the EPO *rs1617640* C-allele is associated with higher expression of the EPO gene and enhanced erythropoiesis. It should be noted that the differences in hemoglobin levels, hematocrit, and red blood cell count between EPO genotype groups were, although statistically significant, rather modest and do not suggest very urgent clinical consequences.

Contrary to our expectations, the EPO *rs1617640* C-allele was associated with an earlier onset of PAD symptoms. Erythropoietin is an angiogenic factor and increased serum erythropoietin levels predicted a good development of collateral vessels in patients with chronic total occlusion of coronary vessels [13,25]. We hypothesize that these potential beneficial angiogenic effects of increased EPO expression may wane by elevated erythropoiesis, resulting in higher blood viscosity and increased risk for microvascular complications.

Furthermore, comparison of tissue-specific gene expression in the Genotype-Tissue Expression (GTEx) Project (https://www.gtexportal.org/) indicated that effects of the EPO *rs1617640* polymorphism could be tissue-specific. In most tissues, including blood and skin, the rs1617640 C-variant was associated with lower gene expression, whereas in other tissues, including prostate and uterus, the C-variant was associated with higher gene expression. These tissue-specific differences might explain the observed associations of the EPO *rs1617640* polymorphism with seemingly contradictory clinical phenotypes.

Two independent studies reported an association of the EPO rs1617640 C-allele with an increased risk for diabetic microvascular complications [17,18]. In contrast to this, Song et al. found no association of the EPO *rs1617640* polymorphism with diabetic retinopathy in a case-control study including 792 individuals with type 2 diabetes [26]. Furthermore, a meta-analysis by Hosseini et al. also showed no association between the EPO *rs1617640* polymorphism and diabetic retinopathy [27]. The reason for this inconsistency is unclear and may involve both different definitions of phenotypes as well as undetected genetic contribution by rare variants in different study populations.

To reduce the risk of a false positive finding, we have restricted genetic analyses of the EPO gene to *rs1617640*, which is currently the only known common EPO variation with functional consequences [14,15]. In other studies, additional EPO polymorphisms, such as rs507392 and rs551238, had no effects on EPO gene expression. Including other EPO polymorphisms would have led to a reduction in prior probability of association and a higher risk of type I (false positive) error [28]. Nevertheless, we are aware that we cannot exclude any effects of rare functional EPO variants on the pathogenesis of PAD.

Furthermore, it should be kept in mind that the present study included patients with prevalent PAD, and data on onset of PAD symptoms were retrieved retrospectively. A prospective study design may provide more precise data of onset of PAD but would require a much higher number of participants and a long follow-up time.

In conclusion, our data suggest that the EPO *rs1617640* gene polymorphism affects erythropoiesis and onset of PAD and is a potential biomarker for the pathogenesis of this disease.

## Figures and Tables

**Table 1:** Demographic and genetic data of peripheral arterial disease (PAD) patients. Age is presented as mean ± standard deviation, all other data are number of subjects (%).

Specifications		PAD Patients (*n* = 945)
Age, years		68.4 ± 10.2
Age at onset of PAD, years		64.8 ± 11.1
Male sex		585 (61.9%)
Ever-smoker		591 (62.5%)
Type 2 diabetes		455 (48.1%)
Arterial hypertension		635 (67.2%)
Hypercholesteremia		653 (69.1%)
*rs1617640* genotype	AA	356 (37.7%) 433 (45.8%) 156 (16.5%)
	AC	
	CC	
*rs1617640* C-allele frequency		0.394

**Table 2:** EPO genotypes and peripheral arterial disease (PAD) parameters.

Specifications	AA	AC	CC	*p*
*n*	356	433	156	-
Age at onset of PAD, years	66.0 ± 10.8	64.1 ± 11.1	64.0 ± 11.5	0.042
Male sex, *n* (%)	227 (63.8)	264 (61.0)	94 (60.3)	0.65
Fontaine stage II, *n* (%) Fontaine stage III–IV, *n* (%)	253 (71.1) 103 (29.0)	295 (68.1) 138 (31.9)	111 (71.2) 45 (28.8)	0.61
Concomitant coronary artery disease, *n* (%)	92 (25.8)	124 (28.6)	41 (26.3)	0.65
Type 2 diabetes mellitus, *n* (%)	167 (46.9)	212 (49.0)	76 (48.7)	0.84
Smoking, *n* (%)	227 (63.8)	265 (61.2)	99 (63.5)	0.74
Hemoglobin, g/dL	13.3 ± 1.9	13.5 ± 1.7	13.7 ± 1.9	0.029
Hematocrit, %	40.3 ± 5.4	41.2 ± 5.0	41.8 ± 5.4	0.006
Red blood cell count, T/L	4.36 ± 0.57	4.47 ± 0.67	4.55 ± 0.59	0.003

Laboratory values for hemoglobin, hematocrit, and red blood cell count were available for 887 (93.9%) patients.

**Table 3:** Multivariate linear regression analysis of age at onset of peripheral arterial disease (PAD).

Risk Factor	Regression Coefficient (95% Confidence Interval), Years	*p*
Male sex (yes/no)	−1.85 (−3.43–−0.7)	0.022
Smoking history (ever/never)	−7.80 (−9.41–−6.18)	<0.001
EPO *rs1617640* (number of C-alleles)	−1.28 (−0.35–−2.21)	0.007
Type 2 diabetes (yes/no)	−0.25 (−1.62–1.12)	0.72
Arterial hypertension (yes/no)	1.72 (0.27–3.17)	0.020
Hypercholesteremia (yes/no)	−1.78 (-3.25–−0.32)	0.017
